# Feasibility and Acceptability of an Internet of Things–Enabled Sedentary Behavior Intervention: Mixed Methods Study

**DOI:** 10.2196/43502

**Published:** 2023-02-27

**Authors:** Yitong Huang, Steve Benford, Benqian Li, Dominic Price, Holly Blake

**Affiliations:** 1 School of Media and Communication Shanghai Jiaotong University Shanghai China; 2 School of Computer Science University of Nottingham Nottingham United Kingdom; 3 School of Health Sciences University of Nottingham Nottingham United Kingdom; 4 NIHR Nottingham Biomedical Research Centre Nottingham United Kingdom

**Keywords:** internet of things, health communication, pervasive media, ubiquitous computing, smart objects, wearable device, behavior change wheel, digital intervention, sedentary behavior, workplace intervention, employee well-being

## Abstract

**Background:**

Encouraging office workers to break up prolonged sedentary behavior (SB) at work with regular microbreaks can be beneficial yet challenging. The Internet of Things (IoT) offers great promise for delivering more subtle and hence acceptable behavior change interventions in the workplace. We previously developed an IoT-enabled SB intervention, called *WorkMyWay*, by applying a combination of theory-informed and human-centered design approaches. According to the Medical Research Council’s framework for developing and evaluating complex interventions such as *WorkMyWay*, process evaluation in the feasibility phase can help establish the viability of novel modes of delivery and identify facilitators and barriers to successful delivery.

**Objective:**

This study aims to evaluate the feasibility and acceptability of the *WorkMyWay* intervention and its technological delivery system.

**Methods:**

A mixed methods approach was adopted. A sample of 15 office workers were recruited to use *WorkMyWay* during work hours for 6 weeks. Questionnaires were administered before and after the intervention period to assess self-report occupational sitting and physical activity (OSPA) and psychosocial variables theoretically aligned with prolonged occupational SB (eg, intention, perceived behavioral control, prospective memory and retrospective memory of breaks, and automaticity of regular break behaviors). Behavioral and interactional data were obtained through the system database to determine adherence, quality of delivery, compliance, and objective OSPA. Semistructured interviews were conducted at the end of the study, and a thematic analysis was performed on interview transcripts.

**Results:**

All 15 participants completed the study (attrition=0%) and on average used the system for 25 tracking days (out of a possible 30 days; adherence=83%). Although no significant change was observed in either objective or self-report OSPA, postintervention improvements were significant in the automaticity of regular break behaviors (*t*_14_=2.606; *P*=.02), retrospective memory of breaks (*t*_14_=7.926; *P*<.001), and prospective memory of breaks (*t*_14_=–2.661; *P*=.02). The qualitative analysis identified 6 themes, which lent support to the high acceptability of *WorkMyWay*, though delivery was compromised by issues concerning Bluetooth connectivity and factors related to user behaviors. Fixing technical issues, tailoring to individual differences, soliciting organizational supports, and harnessing interpersonal influences could facilitate delivery and enhance acceptance.

**Conclusions:**

It is acceptable and feasible to deliver an SB intervention with an IoT system that involves a wearable activity tracking device, an app, and a digitally augmented everyday object (eg, cup). More industrial design and technological development work on *WorkMyWay* is warranted to improve delivery. Future research should seek to establish the broad acceptability of similar IoT-enabled interventions while expanding the range of digitally augmented objects as the modes of delivery to meet diverse needs.

## Introduction

### Background

In the past decade, ample evidence has accumulated to suggest the unfavorable association between sedentary behavior (SB) and cardiometabolic health, even after adjusting for the amount of exercise [1-3]. Moreover, the amount of sedentary time accumulated in single bouts that last longer than 30 minutes (ie, sustained sedentary bouts) and 60 minutes (ie, prolonged sedentary bouts) adds to the risks, whereas breaks in sedentary time are beneficially associated with metabolic biomarkers [3-5]. With a larger proportion of the workforce employed on sedentary occupations, occupational sitting has become a public health concern in modern Western societies. Based on studies with office-based workers in Australia and the United Kingdom (UK), occupational sitting contributed more than half of total sedentary time on workdays [6-9]. Self-report and accelerometer studies have consistently demonstrated that office workers spend most (varying from 60% to 82% across studies) of their working hours on sitting [10-13]; moreover, office workers’ within-work time is characterized by more sustained (12%-34.8% of total sitting) and prolonged (25%-49.8% of total sitting) sedentary bouts with fewer breaks than nonwork time [7,11]. This makes the office-based workplace a priority setting for interventions targeting SB reduction through the promotion of regular break behaviors.

It is challenging to design an intervention that interrupts users at work at opportune moments and encourages them to move around without causing disturbance or annoyance. Internet of Things (IoT) technologies, characterized by ubiquitous sensing, context-aware computing, and embedded interfaces, have shown great promise for delivering just-in-time adaptive interventions to improve health behaviors nonintrusively in everyday settings [14,15], including the workplace [16]. Yet, there is a dearth of theoretically driven development and evaluative work on IoT-enabled health behavior change interventions.

We have previously reported, in detail, the design and development of an IoT-enabled occupational SB intervention called *WorkMyWay* following the Behavior Change Wheel and human-centered design approach [17]. In this paper, we report the next phase of research, namely the “feasibility phase,” under the framework of the UK Medical Research Council (MRC) for developing and evaluating complex interventions [18]. Emphasis will be placed on evaluating the feasibility and acceptability of the intervention process [19].

### Process Evaluation in the Feasibility Phase

While randomized controlled trials of interventions are important to answer questions on the effectiveness and efficacy of the intervention, translation of the evidence into the diverse settings of everyday practice is often challenged by uncertainties in delivery across contexts [20]. This gives rise to the importance of mixed methods process evaluations to answer questions such as how and under what circumstances an intervention can bring about changes [19].

For research involving automated sensors (eg, accelerometer) either for outcome measurement or for delivering just-in-time adaptive interventions, the quality of tracking has great influence on research and intervention feasibility. As demonstrated by Tang and colleagues [21], adjusting for data incompleteness would significantly affect outcome measures and conclusions about behavior change efficacy. In view of this, the occurrence and severity of data loss caused by technological issues and nonadherence should be routinely monitored and considered as indicators of feasibility in this phase. Moreover, process evaluations can explore contexts in which technological failures are more likely to occur, as this will inform the improvement of protocols and development of strategies to minimize the occurrence and adverse impacts of technological failures. Last but not least, considering the potential of analyzing technology-captured data to understand processes of change and identify active intervention ingredients in future larger-scale evaluations [22], it is important to ascertain, at an early stage, whether system data of satisfactory quality can be collected and used for analysis.

Acceptability should be another area of focus in process evaluations in the feasibility phase [19]. Indeed, acceptability is integral to feasibility, because interventions disfavored by participants are unlikely to be implementable in subsequent trials [23]. This is especially the case for digital behavior change interventions, as the quantity and quality of interventions received by a user are dependent on the extent to which the user likes and engages with the digital technology [24,25].

### Objective of This Study

The objective of this study was to assess the feasibility and acceptability of *WorkMyWay* in real-life office settings through examining the following: (1) retention, adherence, compliance, and quality of tracking; (2) participants’ experiences of *WorkMyWay,* including perceived fidelity and quantity of delivery, and contextual factors that would potentially affect the adoption and effectiveness of *WorkMyWay*; and (3) potential for changes in occupational sitting and physical activity (OSPA) and psychological variables theoretically aligned with the hypothesized mechanisms underpinning the intervention.

## Methods

### Study Design

**Figure 1:**
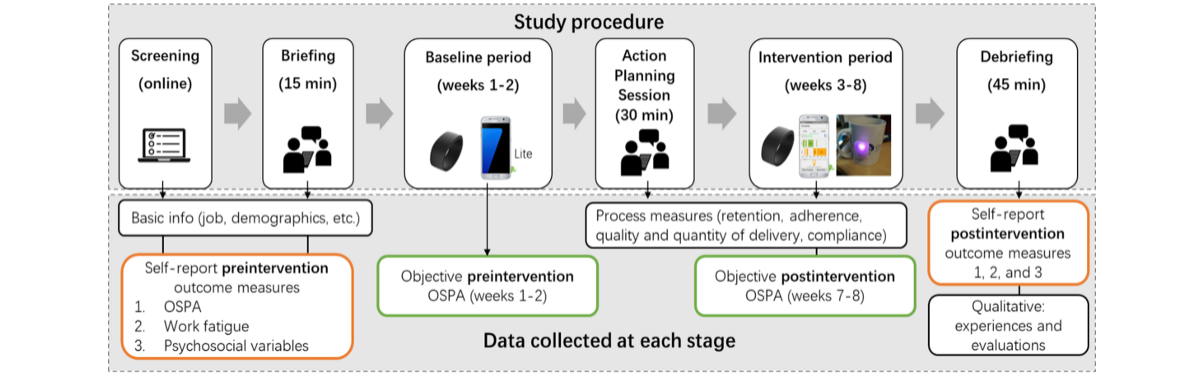
Study procedure and data collected at each stage. OSPA: occupational sitting and physical activity.

This was a mixed methods process evaluation with a single-group pretest-posttest design. Figure 1 visualizes the study procedure and data collected at each stage.

### Ethical Considerations

The study was approved by the Ethics Committee at the School of Computer Science, University of Nottingham (ID 20170920). The information sheet and consent form are enclosed in Multimedia Appendix 1. Study data are all anonymized with individuals represented by participant IDs. A £50 (US $62) Amazon voucher was offered to each participant upon full completion of the study to compensate for their time and feedback.

### Intervention

The *WorkMyWay* intervention was developed in accordance with the framework of the UK MRC for complex intervention research [26], by following through the process of identifying and summarizing the best available evidence [16], developing a theoretical understanding that is likely to account for the process of change [27], theorizing the intervention in terms of the key behavior change techniques and mechanisms, and involving the target recipients and stakeholders of the intervention throughout the development process [17].

The resulting intervention is complex and has been detailed elsewhere [17], using the TIDieR (Template for Intervention Description and Replication) checklist [28]. In brief, the intervention is centered on an IoT system called *WorkMyWay*, which consists of a wrist-worn activity monitor, a light-emitting diode (LED) break reminder attached to the user’s own cup or water bottle, and an Android app that communicates with both devices over Bluetooth low-energy connections. The system uses the movement data livestreamed from the wrist device to detect the user’s period of inactivity in real time and deliver 2 major interventional components.

**Figure 2:**
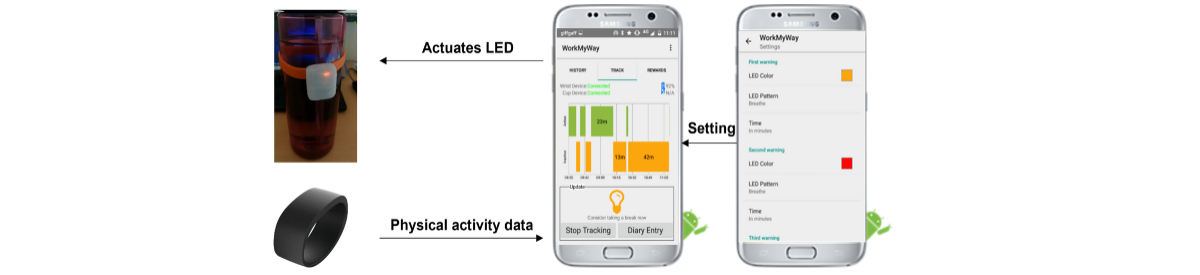
The tracking and prompting component. LED: light-emitting diode.

The first interventional component features quick and actionable point-of-behavior prompts delivered during work hours via the LED device attached to the user’s vessel, an object well-integrated into most office workers’ daily routines with strong associations with work break activities. Based on consultations with stakeholders, the following reminder rules were set as default: if the user is inactive for 45-55 minutes, the cup LED turns into an amber breathing light, meaning “you can consider a break now!”; if the user is inactive for 55–60 minutes, it becomes a red breathing light, meaning “you should take a break now!”; and if the period of inactivity exceeds 60 minutes, it turns into a red flashing light, warning the user of the emergence of a prolonged stationary period (Figure 2).

**Figure 3:**
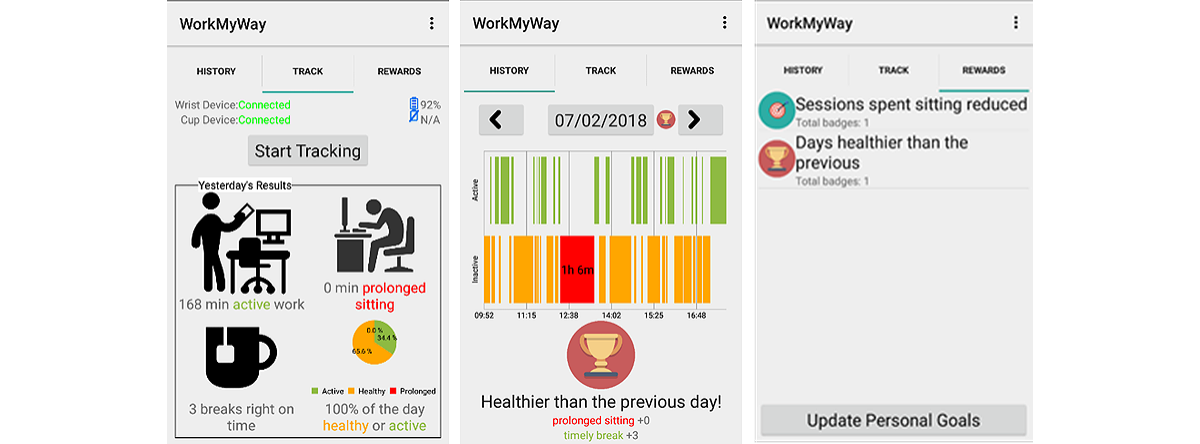
The feedback and reward component.

The second component features more detailed and in-depth feedback and rewards delivered via a screen-based medium (the app) that the user engages with after each workday (Figure 3). Consistent with the LED color scheme and mimicking a traffic light system, the app uses amber, red, and green bars to signify normal inactive bouts (ie, bouts of <60 minutes), prolonged inactive bouts (ie, bouts of >60 minutes), and active breaks (ie, ambulatory bouts), respectively.

Regarding intervention delivery, participants were required to first use a lite version of *WorkMyWay* that only supported tracking while masking all other functionalities from the user for 2 weeks, to obtain baseline SB. This was followed by a 30-minute action planning session where the participant and the researcher (one of the authors) reflected on the baseline data, discussed personal goals, set up action plans, and configured the full *WorkMyWay* system. Afterward, the participant was provided with the full system for another 6 weeks (intervention period). A weekly reminder email was sent to all participants by the researcher on each Monday morning to enhance adherence.

### Sampling and Recruitment

Feasibility studies do not require formal sample size calculation or power calculation [29]. A sample size of 15 is deemed sufficient to uncover most usability and user experience issues [30], which has been used in prior studies to assess feasibility and acceptability of similar eHealth interventions [31-34]. Hence, we recruited a convenience sample of 15 university-employed office workers from 2 local and geographically adjacent workplaces (a university campus and an acute teaching hospital campus) via staff mailing lists and on-campus posters. Potential participants were directed to an online sign-up form with screening questions assessing the following eligibility criteria: (1) no physical disability prohibiting engagement in light physical activity; (2) employed full-time on a job that involved significant amounts of desk-based work (≥50% of total office hours); and (3) normally had the discretion over when to take microbreaks on workdays. Those meeting all the aforesaid criteria were contacted by the researcher to schedule a briefing and consent session in their own offices or a nearby meeting room.

### Quantitative Data Collection and Analysis

#### Overview

We used a combination of system logs and surveys for quantitative data collection. Table 1 summarizes key process and outcome measures calculated based on data accessed from the system and processed using Python (Python Software Foundation), a high-level, general-purpose programming language. The following subsections provide a brief explanation.

**Table 1:** Process and outcome measures calculated based on system data.

Measures		Calculation
**Process measures**		
	Adherence	Tracking days/30
	Quality of tracking	Valid tracking days/tracking days
	Compliance	Prompts with a latency of ≤15 minutes/total prompts triggered
**Objective OSPA^a^**		
	Daily ambulatory time	Accumulated time spent on bouts classified as “active” by the *WorkMyWay* algorithm
	Daily stationary time	Accumulated time spent on bouts classified as “inactive” by the *WorkMyWay* algorithm
	Number of prolonged stationary bouts	Number of stationary bouts that lasted 60 minutes or above for each day
	Duration of prolonged stationary bouts	Accumulated time spent on stationary bouts that lasted 60 minutes or above for each day

^a^OSPA: occupational sitting and physical activity.

#### Process Measures

According to the algorithm we had developed and detailed in a previous article [17], whenever the tracking was on, a period with 0 counts for 40 or more consecutive 15-second epochs (ie, no data for 10 minutes) would be classified as “invalid tracking,” which was likely caused by technological issues or nonwear time; other epochs were all valid tracking time. Tracking days with over 3 hours of valid tracking time and less than 3 hours of invalid tracking time were regarded as “valid tracking days,” whereas the remaining tracking days were classified as “invalid tracking days.” We operationalized each participant’s “quality of tracking” as the percentage of tracking days that were valid (ie, valid tracking days/tracking days × 100%), which was an indicator of technological reliability regardless of the participants’ intention to adhere. Individual adherence was operationalized as the number of tracking days out of a possible 30 days. We also measured each participant’s behavioral compliance with the intervention. For analytic purpose, the onset of the ambulatory or active bout following the prompt event was seen as the response to that prompt, even though the initiation of that break could be irrelevant to the prompts. The time elapsed in between the prompting event and the response was calculated as “response latency” and each individual’s “compliance” was measured as the percentage of prompts responded to with a latency of 15 minutes or less.

#### Outcome Measures

While behavior change outcomes are not the primary focus of process evaluations, the promise for behavior change can still be examined by observing trends of change in outcome measures and especially psychosocial variables theoretically aligned with the intervention [35].

The following outcome measures on objective OSPA for pre- and postintervention periods were calculated based on the system data using the aforementioned algorithm [17]: daily ambulatory time, daily stationary time (ie, any waking behavior done while lying, reclining, sitting, or standing, with no ambulation, irrespective of energy expenditure [36]), and quantities and durations of prolonged stationary bouts (ie, periods of uninterrupted stationary time that were 60 minutes or above).

In addition to objective outcome measures, a survey (Multimedia Appendix 2) was administered at briefing (preintervention) and debriefing (postintervention) sessions to collect the self-report outcome measures reported in Textbox 1.

**Self-report outcome measures collected.**

**The Occupational Sitting and Physical Activity Questionnaire**

Occupational Sitting and Physical Activity (OSPA) [37] was used to obtain self-report OSPA. For comparison with system-captured objective OSPA shown in Table 1, we calculated self-report stationary time by adding up sitting and standing time and calculated ambulatory time by adding up time spent on walking and heavy labor.

**Work Fatigue**

The 3D Work Fatigue Inventory [38], which was used to assess physical, mental, and cognitive work fatigue.

**Psychosocial Determinants of Regular Break Behaviors**

A 7-point Likert-style (1=strongly disagree to 7=strongly agree) scale, which was used to assess psychosocial variables theoretically aligned with the constructs underlying office workers’ sedentary behavior [27]. These included automaticity of regular break behaviors, using items from the automaticity subscale from the Self-Report Habit Index [39]: intention (eg, “I intend to break up sitting with regular micro-breaks throughout the day”), perceived behavioral control (eg, “All things considered, if I wanted to, I could take regular breaks at work”), prospective and retrospective memory of breaks (eg, “I find it difficult to keep track of time when engrossed in work” or “At the end of each day, I have an idea of how much time I’ve spent in prolonged sitting in total”), and organizational culture (eg, “The organizational culture and climate here discourages regular breaks and I feel I’m being watched”).

#### Quantitative Data Analysis

Data on process measures were analyzed with descriptive statistics. Objective OSPA and survey data were imported to SPSS 22.0 (IBM Corp) for inferential statistical analysis. Differences between pre- and postintervention measures were assessed using paired-samples *t* tests, with statistical significance set at .05.

### Qualitative Data Collection and Analysis

A semistructured interview guide (Multimedia Appendix 3) was developed, informed by the MRC guidance for process evaluation of complex interventions [19], which covered the following topics: participant’s perceived quality and quantity of implementation of various intervention components and contextual factors (ie, facilitators and barriers) influencing the engagement with and effectiveness of *WorkMyWay*. All interviews were audio recorded with consent and transcribed in verbatim. Data were analyzed for themes related to feasibility and acceptability of the *WorkMyWay* intervention using a thematic analysis approach [40], which involved familiarization with the data, generating initial codes, searching for themes, reviewing potential themes, defining and naming themes in a code book, final analysis, and write-up. NVivo version 12 (QSR International) was used to facilitate the organization of codes and themes.

## Results

### Study Sample

Table 2 presents the characteristics of the sample.

**Table 2:** Baseline characteristics of the study sample (n=15).

Characteristic		Value
Age (years), mean (SD; range)		40.5 (11.0; 25-63)
**Gender, n (%)**		
	Male	3 (20)
	Female	12 (80)
**Highest education level completed, n (%)**		
	University preparatory degree	2 (13)
	Undergraduate degree	6 (40)
	Postgraduate degree	7 (47)
**Self-reported occupational time spent (hours), mean (SD; range)**		
	Sitting	6.2 (1.5; 2.4-8.2)
	Standing	0.9 (1.3; 0-4.8)
	Walking	0.8 (0.6; 0.145-2)
	Heavy labor	0.1 (0.5; 0-1.9)
	Total	8.0 (0.9; 7.25-10)
Height (cm), mean (SD; range)		169.3 (7.5; 155-180)
Weight (kg), mean (SD; range)		72.0 (13.6; 49-90)
**BMI (kg/m^2^), mean (SD; range)**		25.0 (4.1; 18.4-33.0)
	Underweight (≤18.5), n (%)	1 (7)
	Normal (18.5-24.9), n (%)	5 (33)
	Overweight (25-29.9), n (%)	8 (53)
	Obese (≥30), n (%)	1 (7)
**Number of officemates, n (%)**		
	0	5 (33)
	1	2 (13)
	3	5 (33)
	>3	3 (20)

### Quantitative Results

#### Adherence and Usage

**Figure 4:**
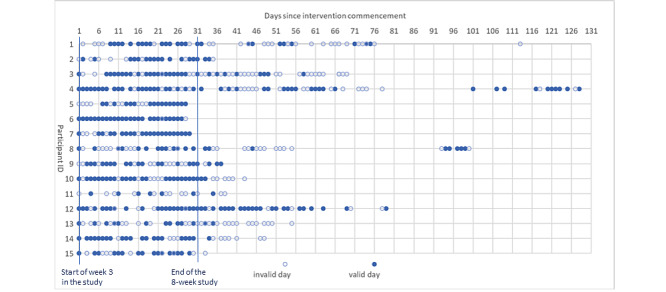
Usage pattern of the WorkMyWay full version.

All participants completed the 8-week study protocol (100% retention), including all measurement and interventional components. Figure 4 provides an overview of the usage data since the installation of *WorkMyWay* full version. Weeks 1 and 2 (ie, the baseline period) were excluded from the graph, as the lite version of the app was used during that period.

The number of tracking days over the intervention period ranged from 15 to 30 workdays across participants, with a mean of 25 (SD 4) days and a median (25th-75th percentile) of 26 (23-28) days. This meant that the adherence rate ranged from 50% (15/30) to 100% (30/30) across participants, with a mean adherence rate of 83.3% (SD 14%) and a median (25th-75th percentile) of 86.7% (76.7%-93.3%).

Of the 375 total tracking days, 262 (69.9%) were valid tracking days. On those valid days, daily valid tracking time ranged from 182.75 to 632.25 minutes, with a mean of 414.2 (SD 94.6) minutes, or 6.9 (SD 1.6) hours; daily invalid tracking time ranged from 0 to 179.5 minutes, with a mean of 23.35 (SD 37.6) minutes and a median of 0 minutes. Anecdotal reports suggested that invalid tracking was mostly caused by data loss during Bluetooth disconnection, which will be detailed in the “Qualitative Results” section.

The number of valid days tracked over the intervention period ranged from 6 to 26 days across participants, with a mean of 17.5 (SD 5.3) valid tracking days and a median (25th-75th percentile) of 16 (14.5-21.5) days. This yielded a mean quality of tracking of 68.6% (SD 14.9%), with a median (25th-75th percentile) of 71.4% (59.3%-81.1%).

After the completion of the 6-week intervention, we offered the option for participants to keep using *WorkMyWay*; 11 (73%) participants opted in to continue using the devices in their own interests, but 2 of them (P6 and P9) had to stop earlier than they would like to because we ran out of devices for new participants. The main reasons for not opting in (P2, P5, P7, and P15) to poststudy use were (1) leaving the university for a new job (n=1), (2) having technical difficulties setting up (n=2), and (3) physical discomfort wearing the wristband (n=2).

Among the remaining 9 participants (P1, P3, P4, P8, and P10-P14) who could use the devices freely for as long as they wanted, the last of day of use (number of days since the study end) ranged from 8 (P11) to 98 (P4), with a median of 39 and a mean of 44.8 (SD 32.5). Self-directed use after the 6-week intervention generated a further 211 days of tracking and usage data, of which 91 days were valid. As expected, poststudy adherence (mean 55.8%, SD 19.3%) and quality of tracking (mean 35.7%, SD 5.4%) were significantly lower than within-study adherence (mean 81.5%, SD 15.3%) and quality (mean 67.3%, SD 5.4%), confirmed by paired-samples *t* tests (*t*_8_=3.619; *P*=.007 for adherence; *t*_8_=4.3; *P*=.003 for quality of tracking).

#### Prompts Delivery and Compliance

A total of 698 time stamped prompting events were recorded. This meant that each participant would have received 1.8 (SD 1.1) prompts on a typical tracking day. The number of prompts received by each participant over the study period ranged from 13 (P11) to 116 (P3), with a median of 37.

**Figure 5:**
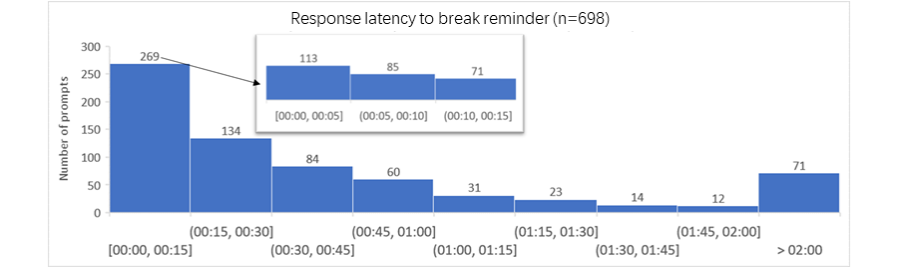
Latency of responses to LED (light-emitting diode) prompts.

As Figure 5 shows, slightly over one-third of the prompts (n=269, 38.5%) were responded to within 15 minutes. Within this category, the majority were responded within 5 minutes (n=113, 16.2%), followed by 5-10 minutes (n=85, 12.2%) and 10-15 minutes (n=71, 10.2%).

#### Promise for Change

As Table 3 shows, there was no statistically significant pre-post difference in any of the behavioral outcomes. However, postintervention improvements were significant in several psychosocial variables theoretically aligned with the target behavior, namely, automaticity of microbreak behaviors (*t*_14_=2.606; *P*=.02), retrospective memory of breaks (*t*_14_=7.926; *P*<.001), and prospective memory of breaks (*t*_14_=–2.661; *P*=.02).

**Table 3:** Behavioral and psychosocial outcome measures at baseline and postintervention (n=15).

Measures		Preintervention, mean (SD)	Postintervention, mean (SD)	Trend (mean difference)	*t* value (*df*)	*P* value
**Objective OSPA^a^ based on tracking data (based on valid days)**						
	Valid tracking time, min/workday	430.4 (45.2)	419.7 (51.4)	–10.7	–0.627 (14)	.54
	Daily stationary, minutes/workday	355.0 (57.3)	356.7 (56.3)	1.7	0.115 (14)	.91
	Daily ambulatory, minutes/workday	75.4 (45.9)	63.0 (28.7)	–12.4	–1.288 (14)	.22
	Duration of prolonged stationary bouts, minutes/workday	176.1 (78.7)	188.3 (95.3)	12.1	0.591 (14)	.56
	Number of prolonged stationary bouts, n/workday	1.8 (0.8)	1.8 (0.7)	–0.05	–0.252 (14)	.80
**Self-report OSPA**						
	Work time, minutes/day	482.5 (55.7)	492.5 (77.5)	10.1	0.569 (14)	.58
	Siting, minutes/day	369.0 (91.1)	373.3 (78.8)	4.3	0.209 (14)	.84
	Standing, minutes/day	56.0 (77.9)	58.6 (61.2)	2.6	0.138 (14)	.89
	Walking, minutes/day	49.5 (38.7)	60.3 (50.6)	10.8	1.131 (14)	.28
	Heavy labor, minutes/day	7.9 (29.4)	0.29 (1.1)	–7.6	–0.998 (14)	.34
**Determinants of breaks**						
	Intention to take regular work breaks	6.07 (0.89)	6.20 (0.86)	0.13	0.695 (14)	.49
	Positive outcome expectancy	6.18 (0.75)	6.27 (0.63)	0.08	0.673 (14)	.51
	Perceived behavioral control	6.20 (0.78)	6.33 (0.82)	0.13	0.487 (14)	.63
	Perceived barrier: heavy workload^b^	5.07 (1.9)	5.00 (1.91)	–0.07	–0.163 (14)	.87
	Perceived barrier: discouraging organizational culture^b^	1.80 (0.561)	1.80 (0.941)	0.00	0.000 (14)	>.99
	Perceived facilitator: organizational culture encouraging breaks	6.00 (1.00)	6.07 (0.80)	0.07	0.202 (14)	.84
	Regular microbreak habit (automaticity subscale)	4.41 (0.71)	4.85 (0.44)	0.43	2.606 (14)	.02^c^
	Retrospective memory of breaks	3.47 (1.47)	6.30 (0.80)	2.83	7.926 (14)	<.001
	Difficulty with remembering to take breaks (prospective memory)^b^	5.70 (1.07)	4.93 (0.92)	–0.77	–2.661 (14)	.02^c^
**Work fatigue**						
	Physical fatigue	2.14 (0.64)	2.05 (0.60)	–0.08	–0.807 (14)	.43
	Mental fatigue	2.69 (0.96)	2.61 (0.86)	–0.07	–0.504 (14)	.62
	Cognitive fatigue	1.57 (0.54)	1.78 (0.52)	0.21	1.809 (14)	.09

^a^OSPA: occupational sitting and physical activity.

^b^Factors with supposedly adverse impacts on regular break behaviors.

^c^*P*<.05.

### Qualitative Results

#### Overview of Themes

A total of 6 themes were identified through the qualitative analysis. These encompass acceptability of tracking, causes of inaccuracy and solutions, barriers to prompts delivery, mixed attitudes toward the embedded medium for delivering prompts, organizational climate and job characteristics affecting intervention uptake, and interpersonal influences on adherence and compliance. Table 4 presents all themes and subthemes with illustrative quotes. The following subsections provide a brief explanation.

**Table 4:** Key themes and subthemes emerging from qualitative analysis with illustrative quotes.

Themes and subthemes		Illustrative quotes
**Theme 1: Acceptability of tracking**		
	Ease of integration into everyday routines	*I think it’s really quite simple to use. You just start and stop. That's how it's supposed work, start tracking and stop tracking.* [P2] *Pretty easy. I guess I have a set-up routine when I get into my office anyway, get my laptop out, set up.* [P4]
	Difficulty with remembering to stop tracking	*I had no trouble coming in every day and turning it on, but I had a couple of days on which, I went back home with my wrist on me. I was like 'no!'...Once you clicked 'tracking' you forget about it.* [P8]
	Discomfort of wearing	*It just gets sweaty and in a way it’s quite annoying.* [P14]
	Accuracy of tracking	*I think like 90% of the time it was accurate in telling whether I’m active or not.* [P10]
**Theme 2: Causes of inaccuracy and solutions**		
	Inaccuracy caused by data loss	*I take my phone when I’m out of the office. But if we just went to the corridor, it was okay to just leave the phone in the office. Sometimes I don’t think it’s recorded things like going to the printer and back from the printer for like 10 or 11 times. I don’t think it had, because it kept saying ‘not connected’.* [P13]
	Reducing data issues with system updates	*They seem really accurate, especially after one update, I can’t remember when it was I updated it. After then it felt really was picking up everything. So I felt like it was quite accurate.* [P15]
	Inaccuracy related to individual differences and needs	*I realized it was quite sensitive because a lot of the stripes were just 1 min. Initially I sat there and thought I haven’t been out of the office. What is it recording? Then I thought, oh, I’ve opened the blind, I’ve got up and put something in the bin. Maybe actually I haven’t physically moved. Then I thought it’s logging that I’m typing.* [P7]
	Adjusting detection thresholds upon individual requests	*I talked to you, if you remember, I had problems with the data not being sent, you restarted it and did something, you also changed the parameters the last time. After that, it was no longer doing that.* [P8]
**Theme 3: Barriers to prompts delivery**		
	Misplacement of the LED^a^ reminder device	*But it's not in a good place on a cup really. It gets in the way. So I tended to use a different cup.* [P5]
	LED facing away from the participants accidentally	*Occasionally I would turn around to look at my bottle and found that I had turned it away from me unconsciously. Then I’ll turn it around and find it flashing.* [P6]
	Not noticing LED flashing in the periphery of attention	*But sometimes when you are concentrating, you don’t really look at things around.* [P13]
	Disconnection between devices	*Although it is there, if it’s not connected for some reason, it doesn’t always light up.* [P13]
**Theme 4: Mixed attitudes toward the embedded medium for delivering prompts**		
	Advantages over vibratory or audible alarms	*I got a Garmin watch that buzzes...This (cup device) was a more subtle way of saying, ‘you need to get up’, as opposed to go out buzzing that’s really disturbing to your surroundings. I really like having the visual cue because I feel like it kind of took my attention away from what I was doing and made me physically look away from what I was doing.* [P11]
	Concerns over disturbance to others	*I’m not sure. I’m in two minds. Coz I was gonna say that it would be useful for me to (have) kind of noise, almost vibrate or buzz or something like that. But if it is 2-hour meeting, and I forget to turn it off, then an hour in, it starts making some annoying noise.* [P15]
	Object cueing and facilitating break activities	*Because it reminds you to do something. You can very well take it as an excuse to fill up your water bottle, or take it and drink it and then fill it up again. It worked for me in that way.* [P3]
	Positive spillover effect on hydration	*It was good to make me drink more rather than just get up, coz it gets me a reason to go to the kitchen and fill my bottle. If it wasn’t attached to a bottle, I might not have taken that with me. I’d just go for a wander. So that was good.* [P14]
	Complexity of managing multiple devices	*Maybe just having one device or one thing embedded in an object that just all works together as one. That'll be much better than having all the individual things.* [P2]
**Theme 5: Organizational climate and job characteristic affecting uptake**		
	Organizational support	*I think this workplace will be happy with it, it's a very flexible department...There is a lot of trust and independent work in timing. I don't think people mind if you get up to go to the bathroom in the middle of a meeting, and things like that.* [P4]
	Job constraints	*But because of the nature of roles, the period of breaks may have to be a bit more controlled. So like student-facing services, they have to be there for particular times, so the breaks are gonna be structured around of their availability and around other’s availability.* [P9]
	Division of responsibilities for employees’ health-related behaviors	*I think the organization doesn’t really mind, or care, either way. They really leave it up to the individuals*...*It would be nice if they would have some options that we could use.* [P12] *As I’m the wellbeing lead, anything that encourages staff to take a practice at work, I’m keen on understanding*...*If you got some summaries of if people actually found it helpful, I’d be quite keen to promote it to university.* [P9]
**Theme 6: Interpersonal influences on adherence and compliance**		
	Subjective norms on regular break behaviors	*It’s a nice environment in that. People are often going out to make a cuppa or asking somebody. Yeah. I think we are all very aware of sitting down all day.* [P10]
	Object triggering social interactions that promoted breaks	*They would go, ‘oh what’s on your water bottle?’ ‘Oh, I’m part of a study’. So, they were interested, and it got them talking. Someone I work with in office could sometimes see the light when she was over at my desk asking me a question or anything, she pointed it out, and we’d be like, ‘oh, maybe we should go get up!*” [P11]
	Office team participation enabled social comparison and social support	*Because we were all in it together. We all had issue. We would sort it out.* [P12] *It was a reward to think, ‘oh yeah, look, I’ve done this this. I showed my colleagues. Have you done this?’ and we compared it.* [P13]

^a^LED: light-emitting diode.

#### Theme 1: Acceptability of Tracking

Most participants reported it was easy to integrate the behavioral tracking into everyday routines and to adhere to the tracking protocol. The email sent by the researcher at the beginning of every workweek was deemed a helpful reminder to recontinue tracking, especially after holidays. Participants found it more difficult to remember to stop tracking at the end of each workday than to start tracking in the morning, because the automated tracking worked unnoticeably at the background throughout the day.

**Figure 6:**
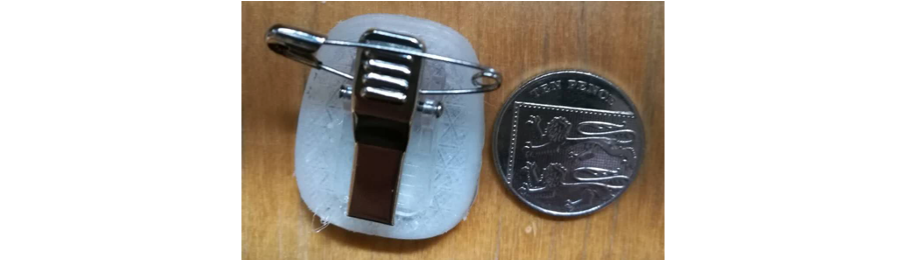
An alternative way of wearing the tracking device suggested by participants.

The discomfort of wearing the wristband (eg, “too tight,” “sweaty in summer”) was identified as a barrier to adherence by participants. As a result, some participants proposed new ways of wearing the “wrist” device using clips, pins, and sellotapes (Figure 6) for poststudy use where more flexibility was allowed in the placement of sensors.

Speaking of the value of tracking, most participants were positive toward the function and thought the algorithm was accurate in differentiating activity (ambulatory behavior) and inactivity (stationary behavior).

#### Theme 2: Causes of Inaccuracy and Solutions

Combining participants’ reports with system logs, perceived inaccuracy occurred mostly during or right after periods of device disconnection when no data were streamed at all. As the *MetaWear* hardware used for the wrist and cup device was supposed to cache data temporarily during short periods of disconnection and resend data to the app upon reconnection, we told participants they need not take the phone with them unless they were out of the office for 15 minutes or longer. However, the devices did not always reconnect as reliably as expected, even after just brief disconnections.

In addition, participants tended to forget to stop tracking and remove the wrist device at the end of each workday, which also caused data synchronization problems the following day. This was due to a flaw in the hardware—with the wrist device logging data in standalone mode for long periods, the microcontroller could be easily overloaded and crashed. Knowing the aforesaid contexts in which data connection problems were likely to occur, we implemented an important system update to make the app automatically clear cache on the MetaWear board if no data were streamed from the wrist device for 10 minutes after first reconnection request. This modification effectively minimized severity of data loss in case of synchronization issues and greatly enhanced perceived accuracy.

Another source of inaccuracy pertained to the need for personalized threshold for activity detection. Some participants reported the algorithm was too sensitive in picking up movements that participants would not consider as breaks (eg, opening the window blind, sitting and talking with hand gesturing). This issue was rectified by adjusting the detection thresholds upon individual requests. We let the participant know upfront that the researcher could adjust the sensitivity of the break detection setting based on each individual’s experience and preference. Three participants (P4, P7, and P8) requested to have the threshold raised so that the break detection became less sensitive.

#### Theme 3: Barriers to Prompts Delivery

Interviews suggested the prompts delivered with the embedded LED (variably called “cup device,” “light” in interviews) did not always reach the participants (ie, low dosage) exactly the way as intended (ie, low fidelity) due to several factors. First, although we had designed *WorkMyWay* to deliver prompts and cues with an object inherently associated with office work breaks (eg, a cup or glass), a few participants did not follow the instruction to attach the LED reminder to vessels that they normally used for everyday hydration. For example, P5, P7, and P9 reported placing the LED device to one vessel while using another vessel for everyday hydration, because the device was “too cumbersome” and “got in the way.” Second, several participants (P4, P6, P14, and P15) reported accidentally putting down the vessel with the LED facing away from themselves. Third, a lack of attentional resources at work to notice the LED flashing in the periphery of attention was reported as another barrier.

In addition to participants’ behaviors, the unreliable connection between the devices compromised prompts delivery.

#### Theme 4: Mixed Attitudes Toward the Embedded Medium for Delivering Prompts

Individual differences existed with respect to the preferred modality and medium of prompting. Some strongly preferred the object-delivered visual prompts to the audible prompts commonly used in commercially available health gadgets, as they thought the LED attached to the object was a “more subtle way” that “effectively directed one’s attention away from they were doing” and “made them physically look away” [P11]. Although some participants did mention vibratory or audible reminders could be more “noticeable,” disturbance to others was raised as a concern.

The idea of integrating prompts and cues for breaks with a break activity–related everyday object was evaluated differently across participants. This approach made a lot of sense and worked well to cue and facilitate breaks for some participants.

In addition, as a positive spillover effect of this medium of delivery, some participants (P1, P2, P3, P12, and P14) reported drinking more liquid. When prompted in interviews, most participants expressed positive attitudes toward the potential addition of technological features to the cup device for tracking, visualizing, and prompting hydration behaviors in the future.

However, several participants reported feeling tired of managing multiple devices, partly because of the unreliable connections between the 3 devices in the current system; a few participants suggested combining the wrist and cup device into 1 to reduce the complexity of system setup.

#### Theme 5: Organizational Climate and Job Characteristics Affecting Intervention Uptake

Organizational support was identified as a major facilitator to the uptake of *WorkMyWay*. All participants in the study thought their employers were happy with the behavioral target (ie, hourly break) promoted by the intervention and permissive of employees’ personal use of technologies as such. However, there were some constraints on break behaviors placed by the nature of the work and the relationships with others involved in the job role.

Different views existed regarding who should be held accountable for employees’ health-related behaviors that occurred in the workplace. Some participants thought the organization and management had “an important role to play.” Although the majority held the view that it should be down to the individual to take care of themselves and to choose the appropriate tools, it would be nice if the organization could offer some options. Encouragingly, one of the participants, who was a senior manager, participated in the study with the interest to source an intervention that could be widely implemented at the university to improve staff well-being.

#### Theme 6: Interpersonal Influences on Adherence and Compliance

The subjective norm, or the perception that a majority in the workplace are trying to take regular breaks, was identified as another facilitator to both using *WorkMyWay* and reducing prolonged SB.

In addition, direct social interactions facilitated the use of *WorkMyWay* most of the time. For instance, when a participant did not notice the LED reminder, there was the chance that coworkers who happened to see the LED flashes could remind him or her. The physical artifact of the technology also turned out to be a conversation piece to get people talking about well-being in the workplace and sometimes to prompt them to take a break together.

For P12, P13, and P14, who shared the same office, participation as an office team enhanced the use and potentially the effectiveness of the intervention through helping each other with troubleshooting, reminding each other to adhere to the study protocol and to comply with the prompts, comparing each other’s data, and competing for fun.

## Discussion

### Principal Findings

This study evaluated the process of delivering *WorkMyWay* in real-life office settings. On the basis of participant experiences, an IoT-based intervention consisting of multiple interconnected devices was complex yet manageable in the workplace. Office workers accepted and adopted the *WorkMyWay* system, as demonstrated with a 100% retention with an 8-week delivery protocol and 83% (25/30) adherence on tracking, which were exemplary for technology-based interventions compared with previous studies [31,41]. Bluetooth disconnection was identified as the major issue impacting on the quality of data and fidelity of delivery, echoing observations from other studies on digital interventions [42]. Nonetheless, this did not deter our participants, as 73% (11/15) continued to use *WorkMyWay* in their own interests after the study ended. While behavior change efficacy is beyond the scope of this study, the significant postintervention improvements in psychosocial determinants of occupational SB suggested high potential for behavior change. Those results together have established *WorkMyWay* as a promising intervention with high potential for long-term adoption and behavior change.

### Potential Intervention Improvements and Broader Research Implications

Our study has revealed various ways in which *WorkMyWay* can be improved as an intervention, which relate to broader implications for the design and delivery of digital interventions targeting SB, and potentially other health behaviors. The first lesson we can draw from the study concerns the importance of designing intervention delivery technologies with minimal reliance on users’ memories. The delivery of the current version of *WorkMyWay* required the user to remember to carry phones even on short breaks, to start and stop tracking on a daily basis, to place the cup device within the field of vision, and to make sure the LED was not facing away from them, which induced uncertainties to the quality and quantity of delivery. From there, we see the need for more engineering work to make data synchronization between different devices more reliable and effortless for the users; we also see a greater role for industrial design in the future to improve the presentation of the LED reminders, for example, by making it an LED ring surrounding a vessel so that it is visible from all directions. These are nontrivial aspects that warrant more considerations and investments in the design and development of digital behavior change interventions.

Second, the study highlights the importance of personalization. Indeed, the ability to dynamically adjust the threshold for activity detection by tweaking parameters in the hidden setting menu was an especially useful feature of *WorkMyWay* and mentioned positively by participants. Personalization should also be supported in the choice and deployment of devices, a need transpired by the fact that participants proposed new ways of wearing tracking devices and placing prompting devices to better fit their individual work practices. This echoes the finding from a previous study that calls for a greater choice of behavior change support tools and devices to be offered to satisfy individualized needs of participants [43]. Despite the proliferation of wearables and IoT technologies, there is a dearth of theoretically informed development of IoT systems for delivering interventions such as the *WorkMyWay*. Another theory-informed intervention most similar to ours is the *Stand More At Work* (*SMArt Work*) intervention [44]. Both interventions feature behavior change techniques such as information about health consequences, prompts and cues, self-monitoring, goal setting, action planning, and feedback on behaviors. Although following broadly similar approaches, the 2 studies embedded prompts and cues into very different everyday artifacts—a cup and a cushion, respectively. One might now ask the question “which of these is the best mode of delivery?” This implies the need for comparative studies of these and potentially other designs before being in a position to roll out an intervention at scale. However, we note an alternative stance, one in which there is no one-size-fits-all intervention design. Rather, interventions may need to be personalized to individuals and contextualized to their particular situations.

Under this view, many potential interventions might be created, for example, by embedding sensors and displays into all manner of everyday objects, tailoring designs to the preferences and contexts of specific individuals. The idea that our interactions with digital technologies should become more personalized and contextualized underlies much research into contextual and ubiquitous computing and its commercial realization in IoT. A third key implication from this research is therefore the need for future research to explore the wider “design space” of possible IoT-enabled behavior change interventions and to deliver generic design guidelines and toolkits for making them (alongside further studies of feasibility and efficacy).

### Strengths and Limitations

A main strength of our study is the mixed method approach that combines system logs, activity tracking data, questionnaires, and interviews to shed light on multiple aspects of the processes of delivering *WorkMyWay*. We demonstrated the feasibility of using technology-captured data to monitor user adherence, compliance, and the quality and quantity of intervention delivery. This approach is advantageous as it allows implementation issues to be considered in relation to the fidelity of individual component delivery in feasibility studies and causal pathways to be potentially modeled in future larger-scale evaluations [22].

Nevertheless, this study has several limitations that should be noted. For instance, the intervention did not sufficiently target the constructs of knowledge and intentions, even though they were considered important determinants of the target behavior [27]. Instead, we decided to place more emphasis on the constructs less explored in previous research (eg, automaticity, prospective memory, and retrospective memory) and target those with sufficient awareness of the issue in the first place by employing self-selection sampling. Therefore, the demographics of the study sample was very different from that of the general population—100% (n=15) of the participants had higher education qualifications, compared with 42% of the UK working population [45]. The demographics of this sample pointed to the possibility of better health-related knowledge and compliance to healthy lifestyle advice than the average population as indicated in previous research [46]. In addition, recruited from higher-education workplace settings, the participants were very supportive of research and tolerant of technological issues, which might not be the case for average office workers employed by other organizations with very different priorities on their agendas (eg, employer targets and financial profit). Therefore, future studies with more representative samples of office workers from a more diverse range of job roles and organizations especially in the private sector are warranted to establish the broad acceptability of *WorkMyWay*.

### Conclusions

It is acceptable and potentially feasible to deliver an SB intervention with an IoT system that involves a wearable activity tracking device, an app, and a digitally augmented everyday object (eg, cup). The findings suggest the interventional contents and technological approach of *WorkMyWay* are viable and it holds great promise to become a successful behavior change intervention. Therefore, it is worth investing in further technological development and industrial design to improve the technology reliability and reduce user burdens. Future research should seek to establish the broad acceptability of similar interventional and technological approaches while expanding the range of digitally augmented objects as modes of delivery to meet diverse needs.

## Appendix

Multimedia Appendix 1 Participant information sheet and consent form.

Multimedia Appendix 2 Questionnaires used for pre- and postintervention assessment.

Multimedia Appendix 3 Debriefing interview guide.

Multimedia Appendix 4 Data set collected and analyzed in the study.

## Abbreviations

IoT: Internet of Things
LED: light-emitting diode
MRC: Medical Research Council
OSPA: occupational sitting and physical activity
SB: sedentary behavior
SMArt Work: Stand More At Work
TIDieR: Template for Intervention Description and Replication

## Data Availability

The system tracking data generated and analyzed during this study are included Multimedia Appendix 4. The questionnaire and interview data are available from the corresponding author on reasonable requests.

## References

[1] de Rezende LFM, Lopes MR, Rey-López JP, Matsudo VKR, Luiz ODC (2014). Sedentary behavior and health outcomes: an overview of systematic reviews. PLoS One.

[2] Bankoski A, Harris TB, McClain JJ, Brychta RJ, Caserotti P, Chen KY, Berrigan D, Troiano RP, Koster A (2011). Sedentary activity associated with metabolic syndrome independent of physical activity. Diabetes Care.

[3] Saunders TJ, McIsaac T, Douillette K, Gaulton N, Hunter S, Rhodes RE, Prince SA, Carson V, Chaput JP, Chastin S, Giangregorio L, Janssen I, Katzmarzyk PT, Kho ME, Poitras VJ, Powell KE, Ross R, Ross-White A, Tremblay MS, Healy GN (2020). Sedentary behaviour and health in adults: an overview of systematic reviews. Appl Physiol Nutr Metab.

[4] Healy G, Dunstan D, Salmon J, Cerin E, Shaw J, Zimmet P, Owen N (2008). Breaks in sedentary time: beneficial associations with metabolic risk. Diabetes Care.

[5] Brocklebank LA, Falconer CL, Page AS, Perry R, Cooper AR (2015). Accelerometer-measured sedentary time and cardiometabolic biomarkers: A systematic review. Prev Med.

[6] Thorp AA, Healy GN, Winkler E, Clark BK, Gardiner PA, Owen N, Dunstan DW (2012). Prolonged sedentary time and physical activity in workplace and non-work contexts: a cross-sectional study of office, customer service and call centre employees. Int J Behav Nutr Phys Act.

[7] Parry S, Straker L (2013). The contribution of office work to sedentary behaviour associated risk. BMC Public Health.

[8] Clemes SA, O'Connell SE, Edwardson CL (2014). Office workers' objectively measured sedentary behavior and physical activity during and outside working hours. J Occup Environ Med.

[9] Bennie JA, Pedisic Z, Timperio A, Crawford D, Dunstan D, Bauman A, van Uffelen J, Salmon J (2015). Total and domain-specific sitting time among employees in desk-based work settings in Australia. Aust N Z J Public Health.

[10] Fountaine CJ, Piacentini M, Liguori GA (2014). Occupational sitting and physical activity among university employees. Int J Exerc Sci.

[11] Ryan CG, Dall PM, Granat MH, Grant PM (2011). Sitting patterns at work: objective measurement of adherence to current recommendations. Ergonomics.

[12] Waters CN, Ling EP, Chu AHY, Ng SHX, Chia A, Lim YW, Müller-Riemenschneider F (2016). Assessing and understanding sedentary behaviour in office-based working adults: a mixed-method approach. BMC Public Health.

[13] Clemes SA, Houdmont J, Munir F, Wilson K, Kerr R, Addley K (2016). Descriptive epidemiology of domain-specific sitting in working adults: the Stormont Study. J Public Health (Oxf).

[14] Sahu KS, Oetomo A, Morita PP (2020). Enabling remote patient monitoring through the use of smart thermostat data in Canada: Exploratory study. JMIR Mhealth Uhealth.

[15] Rose D (2014). Enchanted Objects: Design, Human Desire, and the Internet of Things.

[16] Huang Y, Benford S, Blake H (2019). Digital interventions to reduce sedentary behaviors of office workers: Scoping review. J Med Internet Res.

[17] Huang Y, Benford S, Price D, Patel R, Li B, Ivanov A, Blake H (2020). Using Internet of Things to reduce office workers' sedentary behavior: Intervention development applying the behavior change wheel and human-centered design approach. JMIR Mhealth Uhealth.

[18] Skivington K, Matthews L, Simpson SA, Craig P, Baird J, Blazeby JM, Boyd KA, Craig N, French DP, McIntosh E, Petticrew M, Rycroft-Malone J, White M, Moore L (2021). A new framework for developing and evaluating complex interventions: update of Medical Research Council guidance. BMJ.

[19] Moore GF, Audrey S, Barker M, Bond L, Bonell C, Hardeman W, Moore L, O'Cathain A, Tinati T, Wight D, Baird J (2015). Process evaluation of complex interventions: Medical Research Council guidance. BMJ.

[20] Kessler R, Glasgow RE (2011). A proposal to speed translation of healthcare research into practice: dramatic change is needed. Am J Prev Med.

[21] Tang LM, Meyer J, Epstein DA, Bragg K, Engelen L, Bauman A, Kay J (2018). Defining adherence: Making sense of physical activity tracker data. Proc ACM Interact Mob Wearable Ubiquitous Technol.

[22] Kumar S, Nilsen WJ, Abernethy A, Atienza A, Patrick K, Pavel M, Riley WT, Shar A, Spring B, Spruijt-Metz D, Hedeker D, Honavar V, Kravitz R, Lefebvre RC, Mohr DC, Murphy SA, Quinn C, Shusterman V, Swendeman D (2013). Mobile health technology evaluation: the mHealth evidence workshop. Am J Prev Med.

[23] Michie S, Atkins L, West R (2014). The Behaviour Change Wheel: A Guide to Designing Interventions.

[24] Glasgow RE, Christiansen SM, Kurz D, King DK, Woolley T, Faber AJ, Estabrooks PA, Strycker L, Toobert D, Dickman J (2011). Engagement in a diabetes self-management website: usage patterns and generalizability of program use. J Med Internet Res.

[25] Couper MP, Alexander GL, Zhang N, Little RJA, Maddy N, Nowak MA, McClure JB, Calvi JJ, Rolnick SJ, Stopponi MA, Johnson CC (2010). Engagement and retention: measuring breadth and depth of participant use of an online intervention. J Med Internet Res.

[26] Craig P, Dieppe P, Macintyre S, Michie S, Nazareth I, Petticrew M (2013). Developing and evaluating complex interventions: the new Medical Research Council guidance. Int J Nurs Stud.

[27] Huang Y, Benford S, Hendricks H, Treloar R, Blake H, de Vries P, Oinas-Kukkonen H, Siemons L, Beerlage-de Jong N, van Gemert-Pijnen L (2017). Office workers' perceived barriers and facilitators to taking regular micro-breaks at work: A diary-probed interview study. Persuasive Technology: Development and Implementation of Personalized Technologies to Change Attitudes and Behaviors.

[28] Hoffmann TC, Glasziou PP, Boutron I, Milne R, Perera R, Moher D, Altman DG, Barbour V, Macdonald H, Johnston M, Lamb SE, Dixon-Woods M, McCulloch P, Wyatt JC, Chan A, Michie S (2016). [Better reporting of interventions: Template for Intervention Description and Replication (TIDieR) checklist and guide]. Gesundheitswesen.

[29] Thabane L, Ma J, Chu R, Cheng J, Ismaila A, Rios LP, Robson R, Thabane M, Giangregorio L, Goldsmith CH (2010). A tutorial on pilot studies: the what, why and how. BMC Med Res Methodol.

[30] Nielsen J How many test users in a usability study?. Nielsen Norman Group.

[31] Boulard MC, Martin D, Colombino T, Grasso A (2016). The device is not well designed for me: on the use of activity trackers in the workplace. Proc 12th Int Conf Des Coop Syst.

[32] Pedersen SJ, Cooley PD, Mainsbridge C (2014). An e-health intervention designed to increase workday energy expenditure by reducing prolonged occupational sitting habits. Work.

[33] Mackenzie K, Goyder E, Eves F (2015). Acceptability and feasibility of a low-cost, theory-based and co-produced intervention to reduce workplace sitting time in desk-based university employees. BMC Public Health.

[34] Cooley D, Pedersen S, Mainsbridge C (2014). Assessment of the impact of a workplace intervention to reduce prolonged occupational sitting time. Qual Health Res.

[35] Orsmond GI, Cohn ES (2015). The distinctive features of a feasibility study: Objectives and guiding questions. OTJR (Thorofare N J).

[36] Tremblay MS, Aubert S, Barnes JD, Saunders TJ, Carson V, Latimer-Cheung AE, Chastin SFM, Altenburg TM, Chinapaw MJM, SBRN Terminology Consensus Project Participants (2017). Sedentary Behavior Research Network (SBRN) - Terminology Consensus Project process and outcome. Int J Behav Nutr Phys Act.

[37] Chau J, Van Der Ploeg Hidde P, Dunn S, Kurko J, Bauman A (2012). Validity of the occupational sitting and physical activity questionnaire. Med Sci Sports Exerc.

[38] Frone MR, Tidwell MO (2015). The meaning and measurement of work fatigue: Development and evaluation of the Three-Dimensional Work Fatigue Inventory (3D-WFI). J Occup Health Psychol.

[39] Gardner B, Abraham C, Lally P, de Bruijn GJ (2012). Towards parsimony in habit measurement: testing the convergent and predictive validity of an automaticity subscale of the Self-Report Habit Index. Int J Behav Nutr Phys Act.

[40] Braun V, Clarke V (2006). Using thematic analysis in psychology. Qual Res Psychol.

[41] Lugones-Sanchez C, Sanchez-Calavera MA, Repiso-Gento I, Adalia EG, Ramirez-Manent JI, Agudo-Conde C, Rodriguez-Sanchez E, Gomez-Marcos MA, Recio-Rodriguez JI, Garcia-Ortiz L, EVIDENT 3 Investigators (2020). Effectiveness of an mHealth intervention combining a smartphone app and smart band on body composition in an overweight and obese population: Randomized controlled trial (EVIDENT 3 Study). JMIR Mhealth Uhealth.

[42] Gordon K, Dainty KN, Gray CS, DeLacy J, Shah A, Resnick M, Seto E (2020). Experiences of complex patients with telemonitoring in a nurse-led model of care: Multimethod feasibility study. JMIR Nurs.

[43] Biddle SJH, O'Connell SE, Davies MJ, Dunstan D, Edwardson CL, Esliger DW, Gray LJ, Yates T, Munir F (2020). Reducing sitting at work: process evaluation of the SMArT Work (Stand More At Work) intervention. Trials.

[44] Munir F, Biddle S, Davies M, Dunstan D, Esliger D, Gray L, Jackson B, O'Connell Sophie E, Yates T, Edwardson C (2018). Stand More AT Work (SMArT Work): using the behaviour change wheel to develop an intervention to reduce sitting time in the workplace. BMC Public Health.

[45] Higher Education Statistics Agency (2016). Higher Education Student Statistics: UK, 2016/17 - Qualifications Achieved.

[46] Ross CE, Wu C (1995). The links between education and health. Am Sociol Rev.

