# Supplementary material for: Feasibility and Acceptability of an Internet of Things–Enabled Sedentary Behavior Intervention: Mixed Methods Study
Source: J Med Internet Res. 2023 Feb 27;25:e43502. doi: 10.2196/43502 (PMC10012006; doi:10.2196/43502)
Supplement: Multimedia Appendix 1 [file jmir_v25i1e43502_app1.docx]

## Multimedia Appendix 1. Participant information sheet and consent form.

## Feasibility study of a digital work break intervention -

## Participant Information Sheet

Please take your time and read both the information sheet and consent form carefully before signing. Please retain one copy of this document for your own use. You are encouraged to ask the researcher questions, if anything is unclear.

This study is aimed to evaluate the feasibility of a novel mode of delivering sedentary behaviour interventions to healthy office workers during work hours. The technology only collects data on your physical activity and break habits, but not data relating to any other aspect of your job. No identifiable information about you will be provided to your employer.

This briefing will last approximately 30 minutes. You will be asked some questions about yourself, your work and usual break activities, and factors related to your break patterns at work, based on which the researcher will work with you to set up goals and an action plan that suit you and tailor the technology to best support your goals. Then you will be given an Android smartphone with the study App installed, an activity tracking wristband and a smart LED break reminder. The researcher will give instructions on using the technology and help you fix the LED reminder to the surface of a water bottle or cup you use most in the office. The smart LED will remind you to take breaks based on the minutes of inactivity monitored by your wristband in real time; in addition, you can review how your break pattern has changed over time in the App and achievements.

After Week 2, the researcher will come to see you again. You will have a discussion on your experience of Week 2 and the need to adjust your goals and action plans. Then you will be using the system on your own for another 6 weeks.

After 6-week use of the technology, you will attend a 45-min debriefing interview at a time convenient for you. We will discuss your experience of the technology and study, which will be audio recorded. A £50 Amazon voucher will be emailed to you after the interview to compensate for your time and feedback. You will need to return the research devices. Please keep the study devices and data safe during the study period, just like you would do to your other digital devices and data.

All data transferred to the researcher will be securely stored on the University server only accessible by the researchers involved in the study, in accordance with the Data Protection Act 1998. Your data will be analysed and written up in research publications by the researcher. All data reported in publications will be made anonymised. Any information about you will be described under a pseudonym.

Your participation in this research is entirely voluntary. You may withdraw your consent at any time by contacting the researcher and ask your data collected by that point to be deleted and excluded from the study. During the study, if there is any period in which you don’t want your physical movements to be recorded, simply remove the wristband for the period and put it back on afterwards. If you decide to discontinue using the system halfway through the study (ie. after Week 4), but would still like to contribute your data collected in the previous 4 weeks to research and attend a debriefing interview, you can still receive a £25 Amazon voucher.

This study has been approved by the Computer Science Ethics Committee (CREC) of the University of Nottingham. The researcher is supported by Horizon Centre for Doctoral Training at the University of Nottingham (RCUK Grant No. EP/L015463/1) and Unilever UK Ltd.

For any inquiries please contact: Yitong Huang (Tel: +44 (0) 7821475752, Email: [yitong.huang@nottingham.ac.uk](mailto:yitong.huang@nottingham.ac.uk), School of Computer Science, The University of Nottingham, Wollaton Road, Nottingham, NG8 1BB)

## Feasibility study of a digital work break intervention –

## Participant Consent Form

This is to confirm that I have agreed to take part in a research study conducted by Yitong Huang. I have read the information sheet provided and I understand what is involved.

I freely give my consent to take part in this study. I am a consenting adult over 18 years old and have no disability or health condition that precludes physical movements. I understand that I have the right to withdraw from the study at any time by contacting the researcher without giving a reason.

I understand that by wearing the wristband and using the App, I consent to sharing data about my physical activity and interactions within the App with the researcher.

I understand that my data collected in this study will be stored under the Data Protection Act 1998 and be used anonymously in publication. I have the right to ask for my data to be removed from potential publication submission up to the point of study write up, although once it is published it can no longer be removed.

*Please read the following statements below and select your response by ticking the appropriate boxes:*

| YES NO | I have received a smartphone on loan for this period. I undertake to ensure that this is kept safely and securely and to return it in good condition at the end of the trial. |
| --- | --- |
| YES NO | I understand that the App together with the connected devices will collect data about my physical activity, cup movement and within-App interactions and upload them to the university server. |

| YES NO | I consent to my debriefing interview being audio recorded and transcribed. |
| --- | --- |
| YES NO | I consent to my questionnaire, interview and App data being analysed and reported anonymously in publications and presentations. |
| YES NO | I consent to my interview quotations, sitting pattern and App activity history being referred to in publications anonymously. |
| YES NO | I am confident that the appropriate management approval, if any is required, is in place for me to contribute time and take part in the study in the workplace. |

Signature of Participant: …………………………………………. Date:

Print Name of Participant:

*I have explained the study to the above participant and he/she has agreed to take part.*

Signature of Researcher: …………………………………………. Date:
