# Supplementary material for: Feasibility and Acceptability of an Internet of Things–Enabled Sedentary Behavior Intervention: Mixed Methods Study
Source: J Med Internet Res. 2023 Feb 27;25:e43502. doi: 10.2196/43502 (PMC10012006; doi:10.2196/43502)
Supplement: Multimedia Appendix 2 [file jmir_v25i1e43502_app2.pdf]

Multimedia Appendix 2. Questionnaires used for pre- and postintervention assessment

Participant ID: _______

Part I.

How many hours did you work in the past 7 days? _____________

During the past 7 days, how many days were you at work? ___________

How would you describe your typical work day in the last 7 days? (This involves only your work day, and does not include travel to and from work, or what you did in your leisure time). Make sure it adds up to 100%

a. Sitting ______%

b. Standing ______%

c. Walking ______%

d. Heavy labour or physically demanding tasks ______%

Part II.

Many people experience a sense of extreme or excessive tiredness during and at the end of the work day. This excessive sense of tiredness is called fatigue and can involve one's physical, mental, and emotional resources. The questions below begin by asking about your experience of physical fatigue, followed by your experience of mental fatigue and emotional fatigue. For each question, check the box that most accurately reflects how often you experience each aspect of fatigue.					
**Physical fatigue** involves extreme physical tiredness and an inability to engage in physical activity. During the PAST 30 DAYS, how often did you …	Never	Occasion ally	About half of the time	More often than not	Everday
1. feel physically exhausted at the end of the workday?	○	○	○	○	○
2. have difficulty engaging in physical activity at the end of the workday?	○	○	○	○	○
3. feel physically worn out at the end of the workday?	○	○	○	○	○
4. want to physically shut down at the end of the workday?	○	○	○	○	○
5. feel physically drained at the end of the workday?	○	○	○	○	○
6. want to avoid anything that took too much physical energy at the end of the workday?	○	○	○	○	○
**Mental fatigue** involves extreme mental tiredness and an inability to think or concentrate. During the PAST 30 DAYS, how often did you …	Never	Occasion ally	About half of the time	More often than not	Everday
7. feel mentally exhausted at the end of the workday?	○	○	○	○	○
8. have difficulty thinking and concentrating at the end of the workday?	○	○	○	○	○
9. feel mentally worn out at the end of the workday?	○	○	○	○	○
10. want to mentally shut down at the end of the workday?	○	○	○	○	○

11. feel mentally drained at the end of the workday?	◯	◯	◯	◯	◯
12. want to avoid anything that took too much mental energy at the end of the workday?	◯	◯	◯	◯	◯
**Emotional fatigue** involves extreme emotional tiredness and an inability to feel or show emotions. During the PAST 30 DAYS, how often did you	Never	Occasionally	About half of the time	More often than not	Everday
13. feel emotionally exhausted at the end of the workday?	◯	◯	◯	◯	◯
14. have difficulty showing and dealing with emotions at the end of the workday?	◯	◯	◯	◯	◯
15. feel emotionally worn out at the end of the workday?	◯	◯	◯	◯	◯
16. want to emotionally shut down at the end of the workday?	◯	◯	◯	◯	◯
17. feel emotionally drained at the end of the workday?	◯	◯	◯	◯	◯
18. want to avoid anything that took too much emotional energy at the end of the workday?	◯	◯	◯	◯	◯

Part III.

19	Over the 6 weeks of using WorkMyWay, have you regularly interrupted sitting with short physical activity breaks at work?<br>*(‘regularly’ means every hour or more frequently; ‘break’ means any interruption in sitting, which might be work-related activity, such as printing)*<br>◯ Yes. I’ve been doing that.<br>◯ No, but I really thought I should do it and was trying to get started<br>◯ No, but I might do it<br>◯ No, I have been satisfied with being sedentary at work

	Please Indicate the extent to which you agree with the following statements:	Strongly disagree	Disagree	Somewhat disagree	Neither agree nor disagree	Somewhat agree	Agree	Strongly agree
20	I intend to break up sitting with regular micro-breaks throughout the day.	◯	◯	◯	◯	◯	◯	◯
21	For me a workday with regular micro-breaks is pleasant	◯	◯	◯	◯	◯	◯	◯
22	For me a workday with regular micro-breaks is productive	◯	◯	◯	◯	◯	◯	◯
23	For me taking regular micro-breaks at work is healthy	◯	◯	◯	◯	◯	◯	◯
24	All things considered, if I wanted to I could take regular breaks at work	◯	◯	◯	◯	◯	◯	◯
25	Heavy workload and tight deadlines impel me to sit and work continuously longer than I would like to	◯	◯	◯	◯	◯	◯	◯
26	The organisational culture and climate here allows flexibility in how I complete work and is supportive of regular micro-break behaviours.	◯	◯	◯	◯	◯	◯	◯
27	The organisational culture and climate here discourages regular breaks and I feel I'm being watched	◯	◯	◯	◯	◯	◯	◯

		Strongly disagree	Disagree	Somewhat disagree	Neither agree nor disagree	Somewhat agree	Agree	Strongly agree
28	**For me, taking regular micro-breaks throughout workdays is something I do automatically.**	○	○	○	○	○	○	○
29	*I find it difficult to keep track of time when engrossed in work, when WorkMyWay functions properly*	○	○	○	○	○	○	○
30	Provided that WorkMyWay functions properly, at the end of each day, I have an idea of how much time I've spent in prolonged sitting in total	○	○	○	○	○	○	○
31	Provided that WorkMyWay functions properly, at the end of each day, I have an idea of how often I have taken breaks	○	○	○	○	○	○	○
32	To take regular breaks, I need better prompts/cues (in addition to what I had access to) that remind me to stand up when I've been sat for too long.	○	○	○	○	○	○	○
33	**Taking regular micro-breaks throughout workdays is something I start doing before I realize I am doing it.**	○	○	○	○	○	○	○
34	**For me, taking regular micro-breaks throughout workdays is something I do without thinking.**	○	○	○	○	○	○	○
35	**Taking regular micro-breaks throughout workdays is something I do without having to consciously remember.**	○	○	○	○	○	○	○

	(Only asked in post-study survey)	Strongly disagree	Disagree	Somewhat disagree	Neither agree nor disagree	Somewhat agree	Agree	Strongly agree
36	Taking a micro-break whenever the LED is glowing is something							
	1) I do regularly	◯	◯	◯	◯	◯	◯	◯
	**2) I do automatically**	◯	◯	◯	◯	◯	◯	◯
	**3) I do without having to consciously remember**	◯	◯	◯	◯	◯	◯	◯
	**4) I do without thinking**	◯	◯	◯	◯	◯	◯	◯
	5) that would require effort not to do it	◯	◯	◯	◯	◯	◯	◯
	6) that belongs to my routine	◯	◯	◯	◯	◯	◯	◯
	**7) I start doing before I realize I am doing it**	◯	◯	◯	◯	◯	◯	◯
	8) I would find hard not to do	◯	◯	◯	◯	◯	◯	◯
	9) I have no need to think about doing	◯	◯	◯	◯	◯	◯	◯
