# Supplementary material for: Feasibility and Acceptability of an Internet of Things–Enabled Sedentary Behavior Intervention: Mixed Methods Study
Source: J Med Internet Res. 2023 Feb 27;25:e43502. doi: 10.2196/43502 (PMC10012006; doi:10.2196/43502)
Supplement: Multimedia Appendix 3 [file jmir_v25i1e43502_app3.docx]

**Multimedia Appendix 3. Debriefing interview guide.**

[I] Implementation [M] Mechanisms [C] Contexts

# Implementation: study motivation & experience

Can you start by telling me how you became interested in the study in the first place?

How would you describe your experience of taking part in the study [I]?

- What do you think of the overall **length** of the study?
- Were you provided with enough **instructions** on what you needed to do?

# Process of change

Did you notice any change in your behaviour or attitude over the course of the study? [M]

**[Present objective data illustrating the change to facilitate discussion]**

- Why not: Barriers🡪 tech problem? Work load? Social?
- Why: Facilitators 🡪 useful components

## Technology experience

Can you list features that found most useful?

- From top of the list: how did you make use of it? [M]

Can you list features that found least useful?

- Why? How could it be improved?
- Tracking and prompting
- Did you use the tracking function whenever you were at work?
- How easy or difficult did you find it to integrate and embed the **tech use** into everyday **routines?** [C]
  - Sideway: impact on work; social barriers/facilitators
- Was it accurate in telling if you are moving or inactive?
- Was the reminder always placed within the field of your view [I]
- On average how many prompts do you notice on a day [I] ?
- How did you normally respond to the reminders [M] ?
- Data feedback:
- Did you review the history? When and how? [I] [M]
- Was the feedback accurate/meaningful/actionable/credible?
- Which form of feedback did you look at most? [M]
- Goal setting:
- How did you find the goal setting function? How often did you review and update it?
- ‘About’ page:
- Have ever read the page in the App that describes research on prolonged sitting and guidelines on how often people should take breaks? [I] [M]

Did you have any problems finding your way around the App? [C]

# Contextual barriers:

Embed it into everyday routine: have you had to make any **adjustments** in your day-to-day life as a result of being part of the study?

Did it cause any **inconvenience**?

## If mention ‘social’, then

Social influences [C][I]: Did your co-workers notice and talk about WorkMyWay?

- How do you think they viewed it?
- Do their opinions matter to you?

Do you think the culture in your workplace would encourage or discourage the implementation of WokrMyWay?

Without naming anyone, can you think of anyone who could have potentially benefited from the intervention? What could have been done better to engage them? [reach and uptake]

## If mention ‘annoying’, then

Can you tell me other situations where (you found it disruptive?)

## General

Could you identify any barriers to using WorkMyWay?

Do you have any suggestions as to how the study and tech could be improved?

Could you identify any barriers to regular breaks that haven’t been addressed by WorkMyWay?

Do you have recommendations for improving the behaviour change aspect?

Is there anything you would like to add?
